# Neurodegenerative Disease Risk in Carriers of Autosomal Recessive Disease

**DOI:** 10.3389/fneur.2021.679927

**Published:** 2021-06-04

**Authors:** Sophia R. L. Vieira, Huw R. Morris

**Affiliations:** Department of Clinical and Movement Neurosciences, University College London, Queen Square Institute of Neurology, London, United Kingdom

**Keywords:** neurodegenerative, autosomal, recessive, carrier, GBA1, GRN, TREM2, EIF2AK3

## Abstract

Genetics has driven significant discoveries in the field of neurodegenerative diseases (NDDs). An emerging theme in neurodegeneration warrants an urgent and comprehensive update: that carrier status of early-onset autosomal recessive (AR) disease, typically considered benign, is associated with an increased risk of a spectrum of late-onset NDDs. *Glucosylceramidase beta* (*GBA1*) gene mutations, responsible for the AR lysosomal storage disorder Gaucher disease, are a prominent example of this principle, having been identified as an important genetic risk factor for Parkinson disease. Genetic analyses have revealed further examples, notably *GRN, TREM2, EIF2AK3*, and several other LSD and mitochondria function genes. In this Review, we discuss the evidence supporting the strikingly distinct allele-dependent clinical phenotypes observed in carriers of such gene mutations and its impact on the wider field of neurodegeneration.

## Introduction

Autosomal recessive (AR) inheritance is an important cause of paediatric disease, in which parents are carriers of single copies of disease mutations but are unaffected by disease. Their children have a 25% risk of inheriting both disease mutations leading to AR disease with risk to siblings but in general not parents or children of affected subjects. The mutations are usually loss-of-function (deletions, splice site, or nonsense mutations) but sometimes non-synonymous mutations may also result in significant protein dysfunction. AR disease is conventionally defined by bi-allelic (homozygous or compound heterozygous) mutations leading to a disease phenotype, with heterozygous mutation carrier status (which may be associated with haploinsufficiency) typically considered benign. Interestingly, there may be survival benefits associated with heterozygote carrier status, particularly in resistance to infectious disease which has been proposed to underlie the higher-than-expected carrier frequency of AR disease gene alleles observed in some populations (1). Resistance to cholera and typhoid fever in individuals who carry cystic fibrosis heterozygote alleles supports this hypothesis (1). However, an emerging prominent theme has changed our view of the biology of heterozygote carrier status and has important implications for genetic counselling and the development of new therapies. Carrier status of predominantly paediatric-onset AR disease may lead to an increased risk of a spectrum of adult-onset neurodegenerative diseases (NDDs) (2, 3).

Studies of families and large population cohorts have revealed that heterozygous and biallelic mutations in the same gene can lead to clinically distinct diseases (2, 3). The extensively studied *glucosylceramidase beta* (*GBA1*) gene exemplifies this principle. Classically associated with Gaucher disease (GD), a rare AR lysosomal storage disorder (LSD), *GBA1* mutations are now known to be a common genetic risk factor for Parkinson disease (PD) (2). The notion that *GBA1*-mutation positive PD clinically, pathologically, and pharmacologically mirrors idiopathic PD triggered a generalised hypothesis of shared genetic and pathophysiological mechanisms behind PD and LSDs (2). Indeed, beyond *GBA1* and other LSD gene variants, there is growing recognition of a more widespread association between rare AR diseases and common NDDs. Research to clarify this has recently gathered significant momentum; mutations in *GRN, TREM2, EIF2AK3*, and several mitochondria function genes (*CLN8, LMBRD1, MPI, MRPS34*, and *MUC1*) determine strikingly distinct allele-dependent clinical phenotypes.

We propose that: (i) this phenomenon is likely to occur frequently; (ii) progress with large-scale genome/whole exome sequencing will uncover further examples, spanning numerous diseases and causative genes, and (iii) annotation with respect to recessive disease enables meaningful variant interpretation. Unbiased approaches are required to identify rare gene variants, typically missed in genome-wide association studies (GWAS) which focus on common (occurring with a minor allele frequency of >1 or 5%) variants. Diverse, transethnic study cohorts are ideal to account for variations in carrier frequencies of AR disease gene alleles. Caution is warranted when presuming pathogenicity of rare variants, many of which may have no effect on protein function and disease risk (4).

In this Review, we explore the overlap between AR disease mutations and later onset NDDs, with key genes selected to showcase this genetic convergence. Facilitating the evolution toward personalised medicine, such a paradigm holds the promise of augmenting pre-existing knowledge of NDD pathogenesis, improved patient stratification in clinical trials and boosting the development of targeted disease-modifying therapies. A greater understanding of this link will enable clinicians to offer the most accurate counselling to carriers of such disease genes. This Review presents multiple strands of evidence to confirm this association, including phenotypic assessments of AR disease patients and obligate carriers, overlapping neuropathological features, animal models, and unbiased case-control genetic analyses. Here, we specifically focus on the interplay between AR LSDs, neuroinflammatory and mitochondrial disorders, and NDD risk in risk allele carriers.

## GBA1

### *GBA1*: The Parallels Between GD and PD

The *GBA1* gene located on chromosome 1q21-22, includes 11 exons and 10 introns. *GBA1* encodes β-glucocerebrosidase (GCase), a lysosomal enzyme involved in sphingolipid metabolism catalysing the hydrolysis of glucosylceramide (GlcCer) into glucose and ceramide (Table 1). Biallelic *GBA1* mutations result in GD, characterised by GlcCer-laden macrophages in visceral tissue and clinical presentations of hepatosplenomegaly, pancytopenia, bone disease, and neurological deficits (5). Former GD classifications comprised 3 types, non-neuronopathic (type 1) and neuronopathic (acute, type 2, and chronic, type 3) (5, 6). However, the notion of a spectrum of GD phenotypes, with varying degrees of severity, is now widely accepted (5).

**Table 1:** Structure and function of genes involved in both AR disease and neurodegeneration.

**Gene**	**Genomic location**	**Gene product**	**Subcellular site of gene product**	**Function of gene product**	**AR disease**
*GBA1*	1q21-22	β-glucocerebrosidase	Lysosome	Hydrolysis of glucosylceramide into glucose and ceramide	Gaucher disease
*SMPD1*	11p15.4	Acid sphingomyelinase	Lysosome and secretory	Hydrolysis of sphingomyelin into ceramide and phosphorylcholine	Niemann-Pick disease (type A and B)
*NPC1*	18q11.2	Niemann-Pick C1 protein	Lysosome and endosome	Cholesterol transport	Niemann-Pick disease (type C1 and D)
*NPC2*	14q24.3	Niemann-Pick C2 protein	Lysosome and secretory	Cholesterol-binding protein	Niemann-Pick disease (type C2)
*GLB1*	3p22.3	β-galactosidase-1	Lysosome	Hydrolyses terminal β-galactose residue from gangliosides, glycoproteins, and glycosaminoglycan	GM1-gangliosidosis (type I-III) Mucopolysaccharidosis type IVB (Morquio)
*HEXA*	15q23	β-hexosaminidase α-subunit	Lysosome	Breakdown of gangliosides	GM2-gangliosidosis Tay-Sachs disease
*HEXB*	5q13.3	β-hexosaminidase β-subunit	Lysosome	Breakdown of gangliosides	Sandhoff disease
*GLA*	Xq22.1	α-galactosidase	Lysosome	Hydrolyses the removal of terminal α-galactose groups from glycoproteins and glycolipids	Fabry disease
*ATP13A2*	1p36.13	ATPase 13A2	Lysosome and endosome	Late endosomal/lysosomal P5-type transport ATPase	Neuronal ceroid lipofuscinoses Kufor-Rakeb syndrome
*GRN*	17q21.31	Progranulin	Lysosome and secretory	Lysosomal function and growth factor involved in inflammation, wound healing, and cell proliferation	Neuronal ceroid lipofuscinosis, 11
*TREM2*	6p21.1	Triggering receptor expressed on myeloid cells 2 (type I transmembrane, immunoglobulin receptor)	Plasma membrane (in microglia)	Immune response and inflammation	Nasu-Hakola disease or polycystic lipomembranous osteodysplasia with sclerosing leukoencephalopathy 2
*EIF2AK3*	2p11.2	PKR-like endoplasmic reticulum kinase (PERK)	Endoplasmic reticulum	Ubiquitin protein response	Wolcott-Rallison syndrome
*CLN8*	8p23.3	Transmembrane protein	Endoplasmic reticulum and endoplasmic reticulum-Golgi intermediate compartment	Mitochondrial function Lipid synthesis	Neuronal ceroid lipofuscinosis, 8
*LMBRD1*	6q13	Transmembrane protein	Lysosome	Mitochondrial function Transport and metabolism of cobalamin	Methylmalonic aciduria and homocystinuria, CBLF type
*MPI*	15q24.1-q24.2	Phosphomannose isomerase 1	Cytoplasm	Catalyses the interconversion of fructose-6-phosphate and mannose-6-phosphate Maintains supply of D-mannose derivatives	Congenital disorder of glycosylation, type Ib
*MRPS34*	16p13.3	Mitochondrial ribosomal protein S34	Mitochondria	Mitochondrial ribosomal protein	Combined oxidative phosphorylation deficiency 32
*MUC1*	1q22	Mucin-associated membrane-bound protein	Apical cell membrane	Formation of mucous barriers on epithelial surfaces Mitochondrial function	Medullary cystic kidney disease 1

To date, at least 495 *GBA1* mutations responsible for GD have been identified, encompassing point mutations, insertions, deletions, frameshifts, and recombinant alleles (6). The carrier frequency of *GBA1* mutations is significantly higher among the Ashkenazi Jewish (AJ) population (1 in 14–18) compared to the non-AJ population (<1%) (2, 7). Mutation nomenclature has been recently updated to include the 39-amino-acid leader sequence (newer numbering of the mutated amino acid is shown in parentheses). Five mutations are particularly prevalent, namely p.N370S (p.N409S), p.L444P (p.L483P), IVS2+1G>A, c.84GGIns, and RecNciI (the non-homologous recombination between the functional *GBA1* gene and its pseudogene, *GBAP*) (6). Genotype-phenotype correlations have been performed; N370S (p.N409S) and L444P (p.L483P) homozygosity are mainly associated with mild type 1 GD and severe neurological involvement, respectively (5).

### *GBA1*-Associated PD

Both biallelic and heterozygous *GBA1* mutation carrier status, the latter typically considered benign, confer an increased risk for developing PD. Recognition of this association began in the clinic, with case reports describing parkinsonian symptoms in type 1 GD patients (8), and later in obligate and confirmed *GBA1* carriers (9, 10). Postmortem examinations revealed prominent α-synuclein-positive inclusions (Lewy bodies, LB) in the brains of GD patients and *GBA1* carriers, a pathological hallmark of PD (11, 12). Follow-up large-scale, multicentre analyses confirmed the presence of *GBA1* mutations in 4–15% of PD patients (up to 31.3% in PD AJ cohorts), increasing the lifetime risk of developing PD by up to 20-fold (2). There is no significant difference in PD risk between heterozygous and biallelic *GBA1* mutation carriers (13). For GD-causing mutations, PD risk correlates with the predicted severity of GD: a recent meta-analysis reports that mild and severe *GBA1* mutations have an odds ratio of 2.2 and 10.3, respectively (14). Carriers of severe *GBA1* mutations who developed PD have an earlier age-at-onset and accelerated rates of dementia than those harbouring mild mutations (14, 15). Non-pathogenic *GBA1* polymorphisms in GD patients, p.E326K (p.E365K) and T369M (p.T408M), are also significantly associated with PD, highlight the complexity of the GD/PD relationship (16, 17).

*GBA1*-associated PD (*GBA1*-PD) closely resembles typical sporadic PD. *GBA1*-PD patients exhibit the classic triad of resting tremor, bradykinesia, and rigidity. Distinct characteristics of *GBA1*-PD patients include: younger age of disease onset (by 4–5 years on average); reduced rigidity and an increased frequency of non-motor symptoms, such as cognitive impairment and hyposmia, compared to idiopathic PD patients (7, 18, 19). Of interest, a recent 6-year longitudinal study confirmed increased rates of such prodromal non-motor features in *GBA1* mutation carriers without PD, reporting a significant deterioration in scores for depression, cognition, olfaction, and rapid eye movement (REM) sleep behaviour disorder (RBD) (20). Such prodromal abnormalities are notably identical to those with idiopathic PD. Further follow-up of healthy *GBA1* mutation carriers could enable early diagnoses in those progressing to clinical PD. Recently, unaffected GBA1 carriers have been identified to have microglial activation before the development of overt features of PD (21). The longitudinal clinical and biochemical characterisation of this unique patient cohort may discern biomarkers indicative of PD phenoconversion, prior to significant irreversible neurodegeneration.

**Figure 1:**
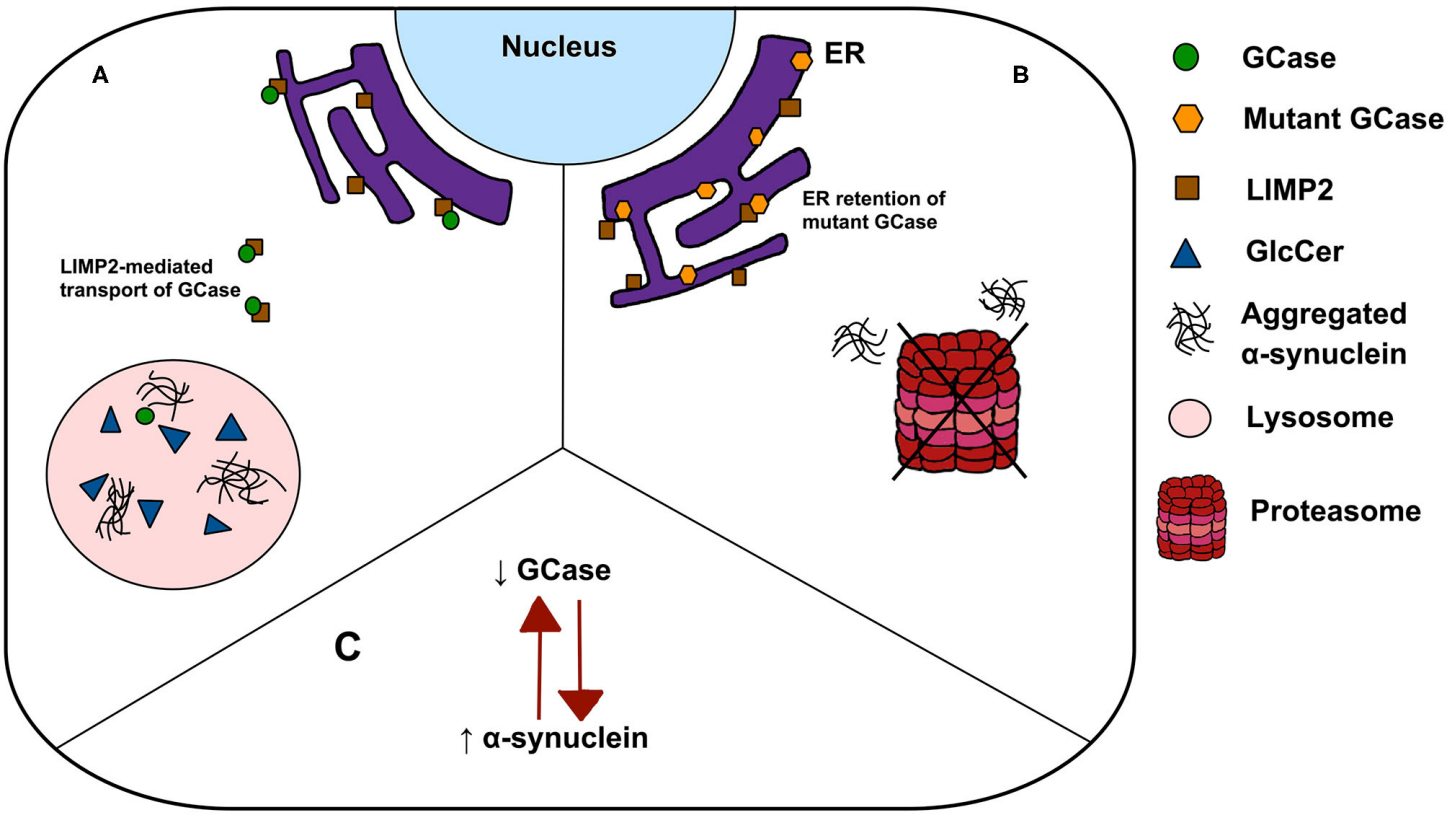
Possible mechanisms linking *GBA1* mutations to PD. **(A)** The loss-of-function theory postulates that In *GBA1* mutation-positive patients, deficient GCase results in GlcCer accumulation, lipid dyshomeostasis and α-synuclein aggregation; **(B)** the gain-of-function model states that ER retention of mutant GCase leads to ER stress, enhancing α-synuclein aggregation and prevent its degradation *via* autophagy or the ubiquitin-proteasome pathway and **(C)** a bidirectional positive feedback loop, in which reduced GCase activity leads to an accumulation of α-synuclein and α-synuclein accumulation further contributes to a decrease in GCase activity.

A major challenge remains to understand the mechanism by which single *GBA1* mutations lead to PD, and how closely this recapitulates the pathogenesis of GD. Several hypotheses have been proposed including: (1) a loss-of-function model characterised by GCase deficiency and its subsequent effect on GlcCer accumulation, lipid homeostasis and α-synuclein degradation, (2) gain-of-function of mutant GCase enhancing α-synuclein aggregation or preventing its degradation *via* autophagy or the ubiquitin-proteasome pathway, and (3) the GCase/α-synuclein bidirectional positive feedback loop, in which reduced GCase activity leads to an accumulation of α-synuclein and α-synuclein accumulation further contributes to a decrease in GCase activity, leading to a self-propagating disease (Figure 1) (22). Interestingly, trafficking of mutant GCase (typically sequestered in the ER) to the lysosome reduced ER stress and reverse locomotor deficits in *Drosophila* with mutant *GBA1* (23), leading to the subsequent analyses of small molecule chaperones in clinical trials (24). Such work highlights the importance of GCase dysfunction in PD pathogenesis, however all proposed pathogenic mechanisms have significant limitations. Null mutations, c.84GGIns, IVS2+1G>A and p.R359X (p.R398T), do not encode the GCase protein but yet have been reported in PD, a finding conflicting with the gain-of-function theory. Similarly, incomplete penetrance of PD in *GBA1* mutation carriers, despite being associated with GCase deficiency, poses a key challenge to the loss-of-function hypothesis. The rate of PD phenoconversion has been reported as 10–30% before age 80, highlighting the notion that GCase deficiency is by no means predictive of PD (25, 26). Further, modifying factors, such as Cathepsin B, play an important role in PD phenoconversion (27). Interestingly, the mutual exclusivity of the aforementioned hypotheses has been challenged. It is possible that the pathogenic pathways may overlap, with evidence indicating that both gain and loss-of-function mechanisms may contribute to PD pathogenesis (28).

### *GBA1* Mutations in Other Synucleinopathies

Synucleinopathies are a diverse group of NDDs characterised by the deposition of inclusions composed of aggregated insoluble α-synuclein, in the form of amyloid fibrils, within the central nervous system. PD, dementia with Lewy bodies (DLB) and multiple system atrophy (MSA) are the most common synucleinopathies. A higher-than-expected *GBA1* mutation burden has been reported among DLB patients compared to control subjects, ranging in frequency from 3 to 34% depending on population heterogeneity, variations in sequencing methods and clinical diagnostic criteria (29, 30). Analyses of neuropathologically confirmed DLB concur with such findings (31). Indeed, a comprehensive, multicentre study revealed an odds ratio of 8.3 for *GBA1* carrier status in DLB, suggesting that the role of *GBA1* mutations in the genetic aetiology of DLB may even surpass that of PD (32). Analogous to *GBA1*-PD, *GBA1*-mutation positive DLB patients demonstrate subtle phenotypic differences compared to their sporadic counterparts, notably an earlier age of onset and greater prevalence of RBD (30). Overall, there is strong support for the notion that *GBA1* mutations exert a large effect on susceptibility for a spectrum of synucleinopathies.

Conflicting evidence has emerged regarding the association between *GBA1* and MSA. Sequencing of *GBA1* coding regions and flanking splice sites in 969 MSA patients (in diverse populations–574 Japanese, 223 European, and 172 North American) revealed a *GBA1* mutation carrier frequency of 1.75% compared to 0.73% in control subjects (33). Albeit modest, such mutations were reportedly associated with the cerebellar variant of MSA, notably a more prevalent clinical variant in Asian populations (33). Further investigations have yielded negative results, indicating no association at all (34, 35). Such findings highlight a distinct pathogenesis for MSA, a disease in which α-synuclein aggregates in oligodendroglial cytoplasmic inclusions rather than neurones. Moreover, preliminary work failed to show an association between *GBA1* mutations and other parkinsonian disorders, progressive supranuclear palsy (PSP) or corticobasal degeneration (CBD), which primarily involve the deposition of tau rather than alpha-synuclein (35). Given the difficulty with genetic power in rare diseases, large-scale multicentre consortia will be required to generate large patient cohorts to fully elucidate the pathogenic contribution of *GBA1* mutations.

### GCase as a Potential Biomarker?

A reliable and validated biomarker is an urgent unmet need in PD to improve diagnostic accuracy, enable accurate disease monitoring and prognostic predictions. Biomarkers for early detection of PD phenoconversion in *GBA1* mutation carriers prior to significant neuronal loss are required for maximal therapeutic benefit. There are inconsistent reports of a decrease in blood GCase activity in *GBA1*-PD and idiopathic PD patients compared to controls (36–39). Interestingly, Cerri et al. (40) found that plasma exosomal/total α-synuclein ratio inversely correlates with GCase activity and disease severity in idiopathic PD patients, replicated in subsequent studies (41). CSF GCase activity is reduced in PD patients independent of their GBA1 mutation carrier status (42). Further, a combination of multiple markers comprising GCase activity has emerged as a more accurate diagnostic tool for PD. Lysosomal markers (GCase, β-hexosaminidase, cathepsin D), total α-synuclein and AD-associated protein Aβ42 demonstrated adequate diagnostic accuracy (sensitivity 84%, specificity 75%) in samples from the PD BioFIND cohort (42). GCase activity, oligomeric/total α-synuclein ratio and age distinguished PD from neurological controls with a sensitivity of 82% and specificity of 71% (43).

### GCase: A Potential Therapeutic Target?

Previously limited to symptomatic therapies enhancing dopaminergic neurotransmission, the discovery of *GBA1* mutations as key aetiological players in PD has driven a novel therapeutic approach. Targeting the GCase/α-synuclein pathway could be significant for *GBA1*-PD patients, asymptomatic *GBA1* mutation carriers at risk of PD phenoconversion and even idiopathic PD patients, where GCase deficiency has also been observed (44).

There are a variety of therapeutic approaches to LSDs particularly GD, including enzyme replacement therapy (ERT), substrate reduction therapy (SRT) and chaperone treatment to enable trafficking of misfolded protein. ERTs are in development and licenced for use in LSDs. If the link between LSD carrier status and late onset NDDs relates to partial enzyme deficiency, enzyme replacement would be a logical and effective treatment/ preventative strategy. However, as outlined above it is not clear that there is a direct relationship between partial enzyme deficiency and late onset NDDs but potentially this may be a future therapeutic approach. Indeed, modulating GCase activity is a focal point for current investigations of disease-modifying therapies in PD.

The inhibitory small-molecule chaperone (SMC), Ambroxol, binds to the active site of mutant GCase, mobilising sequestered mutant GCase from the endoplasmic reticulum (ER) to the lysosome and thus facilitating the normal action of GCase in GlcCer hydrolysis. Promising preclinical data has been reported in two clinical trials assessing the efficacy of Ambroxol in PD and PD dementia patients (ClinicalTrials.gov identifiers: NCT02941822 and NCT02914366, respectively). The Phase IIA prospective, open-label AiM-PD trial reported a modulatory effect on cerebrospinal fluid (CSF) GCase and α-synuclein (24). Notably, two further Phase II trials are due to commence aiming to assess the clinical efficacy of ambroxol on the cognitive, neuropsychiatric and functional outcomes in DLB patient cohorts (ClinicalTrials.gov identifier: NCT04405596 and NCT04588285). Non-inhibitory SMC LTI-291, which modulates GCase post-translational folding by binding to sites other than the active site, is being evaluated in *GBA1*-PD patients by Lysosomal Therapeutic Inc. (Netherlands Trial Register: NTR6960 and NTR7299). Targeting glycosphingolipid accumulation *via* GlcCer synthase antagonists (substrate reduction), a successful treatment for GD, offers an alternative therapeutic strategy in PD. The GlcCer synthase inhibitor, venglustat (GZ/SAR402671), is safe and well-tolerated in *GBA1*-PD patients but did not meet its trial's primary endpoint in PD patients and development has been halted as of January 2021 (ClinicalTrials.gov identifier: NCT02906020) (45). The therapeutic potential of GlcCer synthase inhibitors has been the source of much debate with emerging investigations reporting no sphingolipid accumulation in idiopathic PD and *GBA1*-PD brains (46, 47). Further, GBA1-targeted gene therapy as a means to enhance GCase activity is also under investigation. The ongoing PROPEL study, due to be completed in 2027, is evaluating the therapeutic use of intracisternal administration of gene therapy candidate, PR001A, in *GBA1*-PD patients (ClinicalTrials.gov identifier: NCT04127578).

## LSD GENE VARIANTS

The powerful example of *GBA1* provided the main rationale for broader studies of LSD gene variants in neurodegeneration. Extensive GD registries, notably the International Collaborative Gaucher Group (ICGG) Gaucher Registry, are an invaluable asset, rendering it relatively easy to perform large genetic studies and gather substantial evidence of the *GBA1*-PD association. Due to disease rarity, most studies of non-*GBA1* LSD gene candidates have used small sample sizes, likely being underpowered to detect rare alleles or those with modest effect sizes. The implementation of aggregate burden association tests for joint analyses significantly improves statistical power (48). Employing this framework, Robak et al. (49) revealed an excessive LSD variant burden in PD patients compared to controls. An examination of 54 LSD genes in two large independent cohorts confirmed associations at the *GBA1* and *SMPD1* loci, alongside newly implicating *ASAH1, SLC17A5* and *CTSD* as PD susceptibility genes (49). Biallelic *ASAH1, SLC17A5*, and *CTSD* mutations cause Farber lipogranulomatosis, Salla disease and neuronal ceroid lipofuscinosis (CLN10), respectively. Acid ceramidase (*ASAH1*) is involved in ceramide metabolism, specifically the degradation of ceramide into sphingosine. Further, *CTSD* (Cathepsin D) expression level has been implicated in α-synuclein processing and aggregation (50). Such results recapitulate findings from case reports, reinforcing the importance of lysosomal dysfunction in PD pathogenesis (49).

Multiple investigations of cellular and animal models, as well as neuropathological and phenotypical assessments of patients, point to a genetic parallelism between NDDs and predominantly-AR inherited LSDs. Determining the precise contribution of LSD genes in large population cohorts is crucial. The work by Robak et al. (49) provides evidence for a wide association between LSD genes and PD but more work is needed. Below, we summarise further evidence implicating an association between non-*GBA1*-associated LSDs and NDD, largely focusing on PD.

### Niemann-Pick Disease

Biallelic mutations in either the *SMPD1, NPC1, or NPC2* gene, which are key regulators of sphingolipid metabolism, cause the rare LSD Niemann-Pick disease (NPD). Pathological NPD hallmarks include systemic sphingomyelin accumulation. Extensive phenotypic variability is observed, ranging from psychomotor retardation to visceral organ abnormalities (such as cardiovascular disease and hepatosplenomegaly).

**Figure 2:**
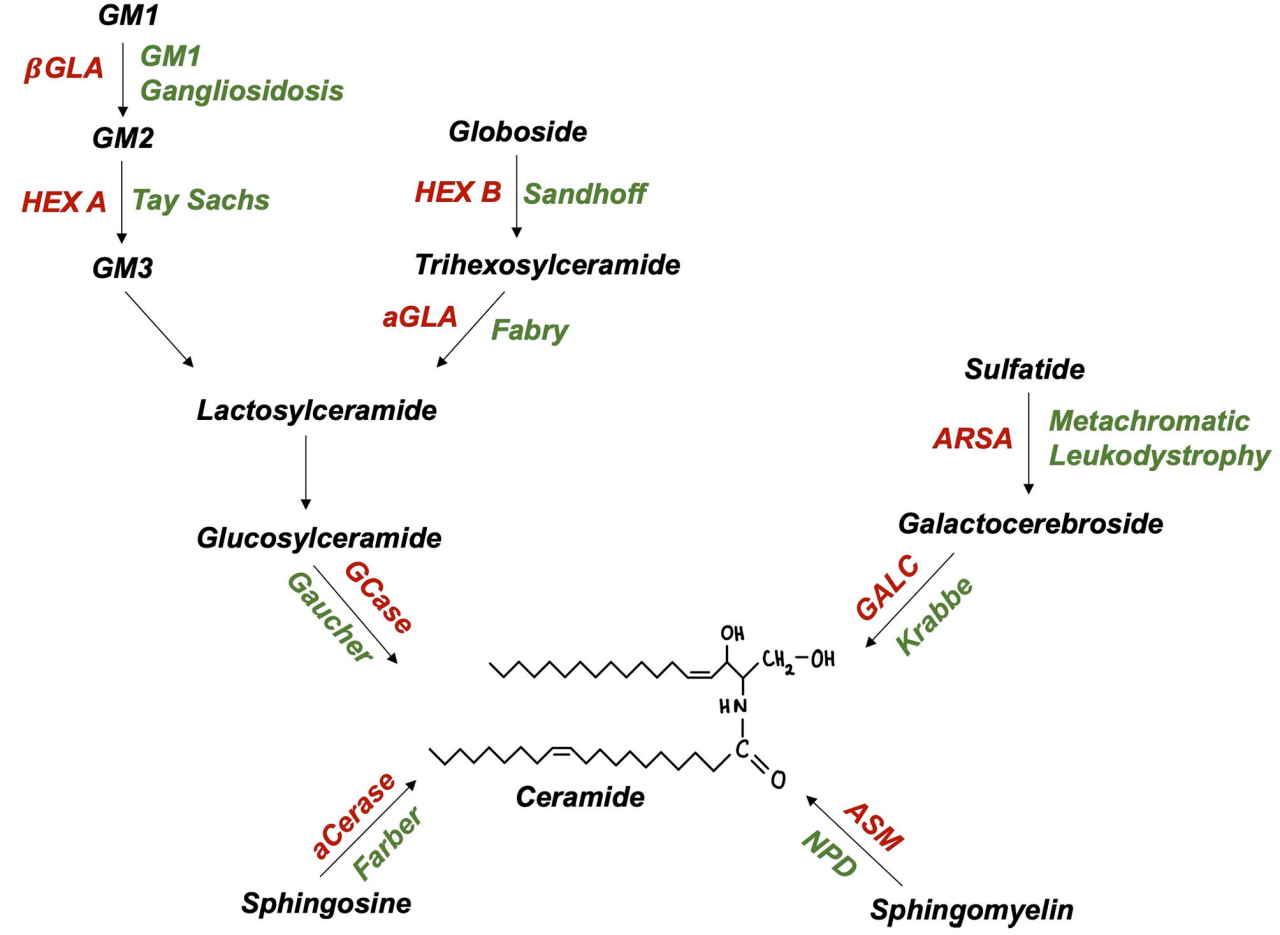
Ceramide and glycolipid metabolism in lysosomes (enzymes in red, autosomal recessive disease in green): aCerase, acid ceramidase; ASM, acid sphingomyelinase; ARSA, arylsulfatase; GALC, galactocerebrosidase; GCase, glucocerebrosidase; aGLA, a-galactosidase; βGLA, β-galactosidase; HEX A/B, hexosaminidase A/B; NPD, Niemann-Pick disease.

The *SMPD1* gene, encoding lysosomal enzyme acid sphingomyelinase (ASM), has recently emerged as a PD susceptibility locus (51). Akin to GCase, ASM hydrolyses sphingolipids to ceramide (Figure 2), suggesting a shared pathogenic pathway to PD. Neuropathological examinations of ASM knockout models revealed significant abnormalities, including: neuronal accumulation of distended lysosomes; cerebellar Purkinje cell degeneration; increased α-synuclein levels and deteriorating motor function (52, 53). Further supporting this link, comprehensive genotyping analyses have identified at least 20 candidate, putative damaging *SMPD1* risk alleles for PD (49, 51). Individual *SMPD1* mutations exhibit differential effects on PD risk, a finding reminiscent of the variable effect of mild vs. severe *GBA1* mutations (51). Following the discovery of over 100 *SMPD1* mutations, an increasing need exists to define PD risk amongst carriers of such mutations (54).

*NPC* heterozygosity may predispose to late-onset neurodegeneration. PD, parkinsonism, atypical parkinsonism (e.g., PSP and CBD), and tremor disorders have been observed in heterozygous *NPC1* and *NPC2* mutation carriers (55–58). Non-motor features including impaired olfaction, urinary urgency, constipation, impotence, orthostatic hypotension, and sleep disturbance were also present (55). Further studies have either failed to support the link between heterozygous *NPC1* mutations and PD (59) or support the notion of its genetic influence beyond movement disorders with reports indicating a pathogenic role in Alzheimer's disease (AD) (60).

### GM1 Gangliosidosis

Biallelic *GLB1* mutations are pathogenic for GM1 gangliosidosis, an AR LSD characterised by β-galactosidase deficiency and ganglioside substrate accumulation within lysosomes (Figure 2). Adult-onset GM1 gangliosidosis patients display increased rates of parkinsonism, ranging from 7.5 to 48% (61, 62). GM1 deficiency was also reported in degenerating dopaminergic neurones of the substantia nigra from idiopathic PD brains (63). The administration of long-term GM1 ganglioside therapy in PD patients resulted in lower rates of symptom progression (64). Indeed, altered GM1-mediated interactions between lipid rafts and α-synuclein may contribute to the formation of pathogenic, oligomeric α-synuclein (65). Further, GM1 ganglioside metabolism imbalances have also been reported in AD and Huntington's disease (66). The rarity of GM1 gangliosidosis and thus, heterozygous *GLB1* mutation carriers, poses a critical challenge to researchers attempting to achieve this and may explain recent failures to find a *GLB1*-PD association in genetic case-control studies (49).

### GM2 Gangliosidosis

Tay-Sachs and Sandhoff disease (GM2 Gangliosidoses) are LSDs caused by biallelic *HEXA* and *HEXB* mutations, encoding lysosomal enzyme β-hexosaminidase α-subunits and β-subunits, respectively (Figure 2). PD-like syndromes, including bradykinesia, tremor, and rigidity have been observed in both GM2 gangliosidosis patients and their relatives (67, 68). Neuropathological examinations revealed enhanced α-synuclein deposition in the brains of GM2 gangliosidosis patients (69). In parallel, PD patients exhibit a reduction in CSF β-hexosaminidase activity (42). A biochemical association between PD and GM2 gangliosidosis is further supported by findings of reduced ubiquitin C-terminal hydrolase (UCH-L1) levels in cultured fibroblasts and brain extracts of Sandhoff disease and disease carrier mouse models (70). Together such pathological evidence highlights the importance of lysosomal function in PD however, investigations of the genetic basis of this association are yet to be conducted.

### Fabry Disease

Recent studies on α-galactosidase A (GLA) deficiency, causing the rare X-linked LSD Fabry disease, suggest there may be an increased risk of developing PD in *GLA* mutation-positive individuals (71). Reduced GLA activity has been reported in dried blood spots and leukocytes collected from idiopathic PD patients compared to healthy controls (72, 73). Stratified analysis by gender revealed no significant difference in GLA activity in male idiopathic PD patients compared to controls, contradicting findings observed in female PD patients (72). Following case reports of parkinsonism in Fabry patients (74), Wise et al. (71) conducted an online survey and family history questionnaire of 90 Fabry patients, finding that 2 patients had received a PD diagnosis (risk of 11.1% by 70 years of age) and a significant family history of PD was present (7.4% had one first degree relative clinically diagnosed with PD). High prevalence rates of PD in late-onset phenotype of Fabry have been subsequently replicated (75). The mechanism by which reduced GLA activity is associated with abnormal α-synuclein aggregation remains unknown.

### Neuronal Ceroid Lipofuscinoses

The neuronal ceroid lipofuscinoses (NCLs) are a heterogenous group of predominantly-AR inherited disorders, characterised by progressive degeneration of the brain and retina. Clinical manifestations are diverse, encompassing retinopathy, epilepsy, motor, and cognitive deficits. Extensive deposition of autofluorescent storage material lipofuscin pathologically defines this disease. With NCL-pathogenic mutations now identified in 13 different genes, an increased understanding of the molecular basis of NCLs has generated compelling evidence of an overlap between altered NCL gene function and NDDs. Highlighting this concept is the recent discovery that biallelic loss-of-function progranulin (*GRN*) gene mutations cause NCL whereas heterozygous loss-of-function mutations cause frontotemporal dementia (FTD, discussed later). Rare variants in the NCL gene *MFSD8* are also candidate risk factors for FTD (76).

Mutations in *ATP13A2* are associated with NCL and Kufor-Rakeb syndrome, a rare juvenile-onset form of PD. Clinical features of parkinsonism are well-documented in the later stages of *CLN3*-associated juvenile NCL (77), showing improvement post levodopa treatment (78). Functional evidence for nigrostriatal pathology, indicative of PD, revealed reduced striatal dopamine transporter density in the putamen and to a lesser extent in the caudate nucleus of juvenile NCL patients (77). Moreover, *Atp13a2*^−/−^ mice exhibit overlapping pathological signatures of NCL (intraneuronal lipofuscin accumulation) and PD (aggregated insoluble α-synuclein) in the hippocampus (79).

## GRN

### *GRN*: The Interface Between FTD and NCL

The *GRN* gene, situated on chromosome 17q21.31, contains 13 exons and encodes the 88-kDa glycoprotein progranulin (PGRN). Ubiquitously expressed, PGRN is implicated in multiple processes including neuroinflammation, tumorigenesis, and lysosome biology. PGRN rose to prominence in the neuroscience community in 2006 following the emergence of two landmark studies causally linking heterozygous loss-of-function *GRN* mutations to familial FTD (80, 81). FTD encompasses a clinically and pathologically heterogenous group of conditions associated with selective atrophy of the frontal and temporal lobes of the brain, comprising the second leading cause of early-onset dementia (82). Interestingly, *GRN* mutations account for 3–26% of familial FTD and perhaps as much as 5% of sporadic cases (82, 83). *GRN* mutations lead to mis-localised TDP-43 pathology, in common with other genetic and sporadic forms of FTD (84).

Since it was first associated with FTD, over 110 pathogenic *GRN* mutations have been described (85). The AD&FTD Mutation Database (http://www.molgen.ua.ac.be/FTDmutations) lists most of these mutations (85). *GRN* haploinsufficiency is thought to underlie *GRN*-associated FTD pathogenesis, with heterozygous *GRN* mutations resulting in a loss-of-function, owing to (1) mutant mRNA clearance by nonsense-mediated decay, (2) prevention of mRNA translation due to missense or splice site mutations of the initiator methionine codon and (3) production of non-functional and unstable protein (81, 86). The net effect is a concomitant reduction of ~50% in PGRN levels, with some estimates at 70–80% (87). Epigenetic modifications may contribute to the lower-than-expected PGRN expression in heterozygous *GRN* mutation carriers (88). GWAS identified lysosomal transmembrane protein 106B (*TMEM106B*) variants as genetic modifiers of PGRN expression (89). Carriers of the *TMEM106B* rs1990622 protective minor allele exhibit elevated GRN plasma levels and delayed age of FTD onset (90), although others have contested such findings (91). *TMEM106B* variants may also account for the age-dependent and incomplete penetrance of *GRN*-associated FTD (*GRN*-FTD), estimated as 50–60% at 60 years and ~90% by age 70 years (81, 92).

Phenotypic characteristics of *GRN-*FTD are highly variable; intrafamilial heterogeneity in clinical presentation is frequently observed (93). *GRN* mutation carriers exhibit considerable variation in age of symptom onset, ranging from 35 to 89 years (94). Consecutive generations of the same family display a >10–20 year difference in age of disease onset (94). Neither disease duration nor age of onset correlate with *GRN* mutation status, type, or location (95). Prominent clinical features of *GRN*-FTD include behavioural variant FTD subtype (bvFTD), significant apathy and social withdrawal (80, 81). Language dysfunction consistent with progressive non-fluent aphasia may occur in the initial stages of disease (93). The occurrence of extrapyramidal features has been noted in ~40% of *GRN*-FTD patients, including dystonia, asymmetric parkinsonism, and CBS with limb apraxia, typically unresponsive to levodopa treatment (93). Further, delusions and hallucinations present more frequently, occurring in up to 30% of FTD patients harbouring *GRN* mutations (96). Characteristic neuroimaging features of *GRN*-FTD brains consist of early parietal involvement, white matter hyper-intensities in atrophied regions and accelerated rates of asymmetric brain atrophy (93, 97). *GRN* mutations are pathologically associated with FTLD-TDP subtype A, characterised by neuronal cytoplasmic inclusions and irregular dystrophic neurites in neocortical layer 2 (84). Notably, TDP-43 inclusions are not specific for FTLD, also being present in motor neurones of amyotrophic lateral sclerosis (ALS) patients, consistent with the FTD-ALS spectrum (82).

Illustrating pleiotropy, while heterozygous *GRN* mutations are pathogenic for FTD, loss of both alleles results in a separate disease entity, NCL with *GRN* designated as CLN11 (3). Interestingly, *GRN* mutations were first associated with a late onset NDD and only several years later with their corresponding AR disease. Indeed, the unanticipated 2012 discovery of homozygous c.813_816del *GRN* mutations in siblings with NCL (3), and later reports of co-occurrence of FTD and NCL within a single family (98), adds further complexity to the *GRN*-FTD association. Subsequent observations of NCL-like pathobiochemical features in FTD patients with *GRN* mutations indicates a greater role of lysosomal dysfunction in FTD pathogenesis (99). The involvement of *TMEM106B* and charged multivesicular body 2B (*CHMP2B*) in FTD, both with key functions residing in the lysosome, strengthen this association (82).

### PGRN: A Potential Therapeutic Target in FTD?

Targeting PGRN haploinsufficiency represents a potential therapeutic avenue in *GRN*-FTD. Identifying presymptomatic cohorts suitable for treatment is facilitated by the specificity and sensitivity of PGRN plasma levels as a predictor of *GRN*-null mutations, and the significant lag (5–10 years) between the onset of neuropathological changes and symptoms (100, 101). Multiple recent trials for FTD disease-modification target PGRN deficiency. Histone deacetylase inhibitor FRM-0334 demonstrated adequate CSF penetration and increased PGRN expression in preclinical models (102). FRM-0334 failed to result in significant changes in PGRN concentrations in 28 subjects with *GRN* mutations in a recent Phase II trial (103), perhaps due to inadequate FRM-0334 exposure (ClinicalTrials.gov identifier: NCT02149160). Evaluations of the safety, tolerability and efficacy of anti-sortilin monoclonal antibody AL001 in *GRN*-FTD patients are underway in a Phase II trial (INFRONT-2, ClinicalTrials.gov identifier: NCT03987295), and as of July 2020, a Phase III trial (INFRONT-3, ClinicalTrials.gov identifier: NCT04374136). Further, the use of calcium-channel blocker nimodipine as a treatment for progranulin insufficiency from *GRN* mutations has been explored (ClinicalTrials.gov identifier: NCT01835665). An 8-week, open-label, dose-finding study revealed no significant alterations in CSF and plasma PGRN concentrations post nimodipine treatment (104). Moreover, alternative approaches under investigation include alkalizing compounds (namely, chloroquine, and amiodarone) and gene therapy to reverse GRN haploinsufficiency (105–107). Multicentre Phase I/II studies are currently investigating the safety, tolerability and efficacy of intra-cisternal adeno-associated viral vector administration of PR006 (PROCLAIM study, ClinicalTrials.gov identifier: NCT04408625) and PBFT02 in *GRN*-FTD patients (upliFT-D study, ClinicalTrials.gov identifier: NCT04747431). Endpoint outcomes include blood and CSF PGRN measurements, with trial completion expected in 2027.

### *GRN* Mutations as Risk Factors for Other NDDs

Heterozygous *GRN* loss-of-function mutations have been confirmed in clinically diagnosed AD patients (108). Indeed, the T allele of single nucleotide polymorphism (SNP) rs5848 located in the 3′ untranslated region of *GRN* particularly contributes to an increased AD risk (109), known to reduce *GRN* mRNA levels in the brain, plasma, and peripheral mononuclear cells (110, 111). Indeed, an inverse correlation exists between AD risk and serum PGRN levels, with AD patients homozygous for the T allele of rs5848 exhibiting the lowest PGRN levels (112). Increased miR-659 binding may underlie such translational inhibition of *GRN* (112). Further, *GRN* mutations were also reported in CBD and ALS, albeit evidence for the latter has been rather inconsistent (113, 114). Large-scale studies, pathological verification of diagnosis and meta-analyses are needed to confirm the wider pathogenic role of *GRN* mutations.

## TREM2

### *TREM2*: A Link Between AD and Nasu-Hakola Disease

AD is a genetically complex disorder, defined clinically by a progressive memory and cognitive decline, and pathologically by extracellular amyloid (Aβ) plaques and intracellular deposits of neurofibrillary tangles (NFTs). Rare, highly penetrant mutations in presenilin-1/2 (*PSEN1/2*), amyloid precursor protein (*APP*) and the ε4 allele of Apolipoprotein E (*APOE*) impart a notable risk for late-onset AD (115). Recent genomic studies revealed rare heterozygous *TREM2* variants as novel genetic risk factors for the disease, offering new keys to decipher AD pathogenesis (115, 116).

*TREM2*, a gene located on chromosome 6p21.1, comprises 5 exons and encodes a transmembrane receptor required for myeloid (or microglial) inflammatory response. To date, over 60 coding *TREM2* variants have been identified, displaying various degrees of population frequency (117). The Alzforum Mutation database provides an up-to-date registry of all potentially pathogenic *TREM2* variants (https://www.alzforum.org/mutations). The most well-studied *TREM2* variant p.R47H (rs75932628) was found to confer a 2- to 4-fold increase in AD risk, a finding independently replicated in numerous North-American and European populations (118, 119). Albeit the p.R47H-AD association failed to be confirmed in several East Asian and other cohorts, perhaps due to diverse population-specific TREM2 allele frequencies (120, 121). Rare *TREM2* variants other than p.R47H are more frequently found in non-Caucasian populations. Reports indicate an increased frequency of novel variants, p.A130V, p.A192T, p.H157Y, and p.S183C in Chinese AD patients (122–124). Allelic heterogeneity was confirmed following the identification of p.W191X and p.L211P variants in African American AD subjects, prompting investigations of different ethnic groups for disease risk variant discovery (120). It is not clear how *TREM2* variants increase an individual's AD risk. *In vitro* studies aiming to elucidate the biochemical effects of such variants report decreased TREM2 stability, altered interactions with lipoprotein ligands and phagocytic functions (125–127). Contrastingly, analyses of AD brains have yielded somewhat inconsistent results, with *TREM2* variants demonstrating no alterations in structure, stability, expression, or ligand affinity (127–129). Nevertheless, *TREM2* variants lead to an impairment of microglial recruitment and response to Aβ plaques, resulting in exacerbation of AD neuropathology. Two loss-of-function mechanisms have been proposed indicating that TREM2 signalling is required to (1) drive the neuroprotective transformation of microglia (130), and (2) sustain microglial cellular energetic and biosynthetic metabolism, allowing their response to stressors (131). Further investigations of *TREM2*-associated AD patient-derived induced pluripotent stem cells and animal models expressing such risk variants should clarify the precise mechanism of the *TREM2*-AD association.

Assessments of the association between particular AD endophenotypes and *TREM2* variants are yet to reach definitive conclusions, hindered by the small sample size of *TREM2* variant carriers. In general, heterozygous *TREM2* p.R47H AD patients typically demonstrate clinical, pathological and neuroimaging features indistinguishable from idiopathic AD (132, 133). An earlier age of AD onset (by ~5 years) and accelerated disease progression have been reported in *TREM2* p.R47H carriers, albeit others contest such findings (132, 133). Other variants within or adjacent to the *TREM* locus, namely *TREM1* intronic variants rs6910730 and rs7759295, may augment (134) or reduce (135) the rate of cognitive decline in AD. Clinically, partial support exists for an increased incidence of psychiatric symptoms and parkinsonian signs, particularly evident in initial stages of the disease (136). Moreover, European AD patients harbouring *TREM2* p.R47H variants display elevated levels of CSF total and phosphorylated tau protein, whereas Aβ42 levels were left unaffected (137).

The mechanism by which biallelic loss-of-function mutations in the same gene *TREM2*, are pathogenic for the very rare Nasu-Hakola disease (NHD), or polycystic lipomembranous osteodysplasia with sclerosing leukoencephalopathy, poses an interesting conundrum. NHD is a rare condition associated with spontaneous fractures, multifocal bone cysts and early-onset dementia. NHD can also be caused by biallelic TYROBP mutations, with reports speculating a role for TYROBP mutations in AD (138). Notably, the *TREM2* R47H variant is associated with AD but not a causative NHD allele, striking similarities with the *GBA1* E326K gene and highlighting mechanistic divergence. Evaluations of several NHD patients by neuroimaging and functional nuclear imaging (^99m^Tc-ECD SPECT) tests revealed key abnormalities including: marked cortical hypometabolism; Aβ deposition in the grey matter of the inferior frontal and occipital lobes, and hypoperfusion of the basal ganglia with concomitant visuospatial memory deficits (139, 140). Immunohistochemistry analyses were less supportive of AD pathology in NHD brains; reports of almost undetectable Aβ plaques and only a small number of NFT-bearing neurones imply that loss-of-function of *TREM2* may not exacerbate AD pathology in NHD (141).

Further work is required to determine how *TREM2* variants influence AD risk and cause NHD. The clinical implications of such variants are potentially significant. Intriguing findings of CSF soluble TREM2 levels as a dynamic marker of microglial activity and thus AD progression, indicate the need to validate its use as a biomarker (142). Additionally, TREM2 may represent a potential therapeutic target in AD. *In vivo* elevation of TREM2 expression confers a rescuing effect, notably promoting microglial survival, reducing amyloid accumulation, and ameliorating memory deficits in AD mouse models (143, 144).

### *TREM2* Variants in Other NDDs

Heterozygous and biallelic *TREM2* variants have been found in FTD cases, particularly bvFTD with atypical presentations, namely seizures (145, 146). A greater severity of TREM2 dysfunction is associated with *TREM2* variants pathogenic for both NHD and FTD: nonsense p.Q33X variant introduces a premature stop codon, halting TREM2 expression; p.T66M and p.Y38C variants, residing in the Ig fold, lead to deficits in TREM2 protein folding, maturation, and expression (145, 146). Initial case-control studies of the association of heterozygous *TREM2* variants as genetic risk factors for FTD in the general population highlighted T96K, L211P, and R47H as significant risk variants (147). Subsequent analyses failed to replicate such findings, prompting the notion that *TREM2* only significantly influences FTD risk at the gene, not variant, level (137).

Findings of *TREM2* variants in other NDDs have been less definitive. The *TREM2* p.R47H variant appears to confer susceptibility to ALS and PD (148, 149). Of note, underlying differences in minor allele frequency for *TREM2* p.R47H in study cohorts across populations may account for the discrepant results generated in attempts to confirm this reported association (150). Further investigations of *TREM2* variant burden in PSP, MSA, and LBD patients have been conducted, yielding inconclusive findings (151–153).

## Other Notable Genes

Emerging findings have implicated numerous genes in AR disease and neurodegeneration (see Table 2 for summary). Additional GWAS of 1,114 PSP cases and 3,247 controls revealed heterozygous *EIF2AK3* rs7571971 common variants, located at the 2p11.2 locus, as significant genetic risk factors for PSP (162). In 2000, mutations in *EIF2AK3* were found to be pathogenic for the rare AR Wolcott-Rallison syndrome, a form of monogenic neonatal diabetes mellitus with concomitant skeletal dysplasia and recurrent hepatitis (163). This discovery highlights intriguing insights into PSP pathogenesis. Encoding PERK, a key regulator of the ubiquitin protein response (UPR), it has been proposed that PERK dysfunction increases ER stress-mediated neuronal damage, ultimately leading to PSP. Indeed, multiple lines of evidence implicate UPR perturbation as a common pathway in NDDs. *EIF2AK3* p.R240H carrier status confers increased AD susceptibility (161), especially in *APOE* ε4-positive subjects (164). Upregulated UPR has also been observed in PD, FTD, and ALS; intriguingly, pharmacological inhibition of PERK signalling is neuroprotective in animal models of such diseases (165–167). Such work supports the need to explore *EIF2AK3* variant burden in a broad range of NDDs.

**Table 2:** Summary table of the main genes associated with AR disease and NDDs (genes with significant evidence supporting association selected).

**Gene**	**Associated disease in**		**Mutation frequency in NDD subjects (%)**	**Odds ratio**	**Typical AR disease features**	**Typical NDD phenotypic features**	**Distinct features of NDD phenotype in mutation carriers vs. non-carriers**	**Gene-targeted therapies in Clinical Trials^a^** (ClinicalTrials.gov Identifier)	**References**
	**Biallelic variant carriers**	**Monoallelic variant carriers**							
*GBA1*	GD	PD	4.2–31.3	5.4	Hepatosplenomegaly Anaemia Thrombocytopenia Bone abnormalities Neurological symptoms	Tremor Bradykinesia Rigidity Postural instability	↑ frequency of NMS in prodromal and PD disease phases Earlier age of onset ↑ frequency and progression of PD motor symptoms Accelerated development of dementia ↓ survival (HR = 1.65)	Ambroxol (NCT02941822) LTI-291 (Netherlands Trial Register: NTR6960 & NTR7299) PR001A (NCT04127578) Venglustat, GZ/SAR402671 (NCT02906020)	(2, 7, 12, 15, 20, 154–156)
		DLB	3.0–34.0	8.3	See above	Progressive cognitive impairment Parkinsonism Visual hallucinations	Earlier age of onset ↑ frequency of RBD Earlier progression to H&Y stage 3	Ambroxol (NCT04588285 & NCT04405596)	(29, 30, 32)
*SMPD1*	NPD	PD	1.7^b^	4.5^b^	Hepatosplenomegaly Thrombocytopenia Pulmonary insufficiency Bone disease Ocular abnormalities Psychomotor deterioration	Tremor Bradykinesia Rigidity Postural instability	Earlier age of onset^c^	–	(53)
*GRN*	NCL	FTD	3.4–25.6 (familial FTD) 1.3–11.7 (total FTD)	3.2^d^	Visual loss Dementia Epilepsy Motor deterioration	Personality change Impaired social conduct Disinhibition Progressive loss of language fluency or comprehension Memory impairment Hyperorality Perseveration behaviours	FTLD-TDP neuropathological subtype A bvFTD subtype ↑ social withdrawal and apathy ↑ language dysfunction in initial stages ↑ hallucinations ↑ parkinsonian features	PR006 (NCT04408625) PBFT02 (NCT04747431) FRM-0334 (NCT02149160) AL001 (NCT03987295 & NCT04374136) Nimodipine (NCT01835665)	(80, 81, 84, 93, 96, 157–160)
*TREM2*	Nasu-Hakola disease	AD	0.6	4.1	Spontaneous bone fractures Multifocal bone cysts Early-onset dementia	Progressive memory and cognitive decline Changes in personality and behaviour	Earlier age of onset Accelerated disease progression ↑ incidence of psychiatric and parkinsonian features	–	(118, 132, 133, 136)
*EIF2AK3*	Wolcott-Rallison syndrome	PSP	61.5^e^	1.8	Neonatal/early-onset insulin-dependent diabetes Skeletal dysplasia Osteoporosis Growth restriction	Postural instability and falls Vertical supranuclear gaze palsy Pseudobulbar palsy Parkinsonism with poor levodopa response	–	–	(161)

*AD, Alzheimer's disease; bvFTD, behavioural variant FTD subtype; DLB, Dementia with Lewy Bodies; FTD, frontotemporal dementia; FTLD-TDP, Frontotemporal lobar degeneration with TDP-43 inclusions; GD, Gaucher Disease; HR, hazard ratio; H&Y, Hoehn and Yahr scale; NCL, neuronal ceroid lipofuscinoses; NDD, neurodegenerative disease; NMS, non-motor symptoms; NPD, Niemann Pick Disease; PD, Parkinson Disease; PSP, Progressive Supranuclear Palsy; RBD, rapid eye movement sleep behaviour disorder; UPR, unfolded protein response*.

a *From ClinicalTrials.gov unless noted otherwise. Accessed April 2021*.

b *Data from Alcalay et al. (53) of combined analyses of previously published AJ populations, notably healthy AJ controls and NDD patients carrying p.L302P and p.fsP330 SMPD1 mutations*.

c *Analyses by Alcalay et al. (53) revealed that among PD patients, reduced ASM activity was associated with earlier age of PD onset (significant difference of 3.5–5.8 years)*.

d *The odds ratio to develop FTLD-ubiquitin positive inclusions for homozygous carriers of minor T-allele of rs5848 GRN variant compared with homozygous C-allele carriers*.

e *EIF2AK3 carrier frequency in the presence of at least one copy APOE ε4. ↑ An increase in; ↓ A decrease in*.

More comprehensive understanding of the genetics of mitochondrial disease revealed new heterozygous gene variants, involved in mitochondria function, to be associated with PD risk and later age of disease onset (168). Robust evidence implicates the genes *CLN8, LMBRD1, MPI, MRPS34*, and *MUC1*, pathogenic for the following AR diseases, NCL8, Methylmalonic aciduria and homocystinuria, congenital disorder of glycosylation type Ib, Combined oxidative phosphorylation deficiency 32 and Medullary cystic kidney disease 1, respectively (168). It is likely further unbiased analyses may yield more genomic associations between NDDs and rare AR diseases; therapeutics targeting mitochondrial processes may be beneficial in the initial stages of PD.

## Conclusions

Emerging evidence presented in this Review highlights a recurring theme of pleiotropic effects of biallelic and heterozygous gene mutations. Carrier status of several AR diseases, hitherto perceived as benign, are now known to confer increased NDD risk. The advent of widespread implementation of high throughput sequencing will certainly unravel further examples. Caution is required when implicating pathogenicity to the presence of *de novo* gene variants identified *via* this approach; certain variants may indeed be neutral in the general population. It is important that future case control studies in NDD incorporate our progress in understanding rare AR diseases. Well-designed case control studies will include careful matching of cases and control populations (given the variation in underlying rare allele mutation frequency) and the variants will be annotated with respect to their known AR disease biology. Annotation of rare disease gene variants is an important aspect of evaluating carrier status, clinical counselling, and attributing functional impact to variants. These may be distinct, so for the case of *GBA1*, two non-GD causing variants, p.E326K (p.E365K) and T369M (p.T408M), are associated with PD. It may be difficult to interpret or predict the effect of rare non-synonymous variants, as seen in GD, knowledge of their effects in AR disease may be helpful and clinical variant databases such as ClinVar may be of use here. Transethnic studies may also be important with different alleles in different populations being associated with disease.

Improved understanding of the genetic basis of NDDs creates opportunities to better elucidate disease pathogenesis and develop targeted therapeutics for genetically, and thus molecularly, defined subsets of patients. In particular, further research is required to ascertain whether the search for treatments for AR disease will benefit carriers of AR disease genes presenting with adult-onset NDDs. Substrate reduction *via* GlcCer synthase antagonists is one such example, already having been established as a successful therapy for GD (45). The known biology and biochemistry of AR diseases provides new insights into late onset NDD. However, the spectrum and effect of variants may be different in AR disease and NDD, as seen in *GBA1* and *TREM2* variants with NDD associated alleles may not be pathogenic for AR disease, highlighting mechanistic divergence. Moreover, insights gained in exploring the association between AR disease and NDDs adds greater complexity to genetic counselling, enabling heterozygote carriers of AR disease genes to be fully informed of their risk for adult-onset NDDs. We hope that research in this area will benefit both adults and children suffering from these progressive degenerative conditions.

## Author Contributions

SV: conception, organization, and execution of the research project and writing of first draft, review, and critique of the manuscript preparation. HM: conception and organization of the research project and review and critique of the manuscript preparation. Both authors contributed to the article and approved the submitted version.

## Conflict of Interest

HM is employed by UCL. In the last 24 months he reports paid consultancy from Biogen, Biohaven, Lundbeck; lecture fees/honoraria from Wellcome Trust, Movement Disorders Society. Research Grants from Parkinson's UK, Cure Parkinson's Trust, PSP Association, CBD Solutions, Drake Foundation, Medical Research Council, Michael J Fox Foundation. HM is a co-applicant on a patent application related to C9ORF72—Method for diagnosing a neurodegenerative disease (PCT/GB2012/052140). The remaining author declares that the research was conducted in the absence of any commercial or financial relationships that could be construed as a potential conflict of interest.
